# A modified decontamination and storage method for sputum from patients with tuberculosis

**DOI:** 10.12688/wellcomeopenres.18888.1

**Published:** 2023-04-13

**Authors:** Nguyen Le Quang, Do Dang Anh Thu, Le Pham Tien Trieu, Nguyen Hong Hanh, Nguyen Huu Lan, Dang Thi Minh Ha, Guy Thwaites, Nguyen Thuy Thuong Thuong, Timothy M. Walker

**Affiliations:** 1 Oxford University Clinical Research Unit, Centre for Tropical Medicine, Ho Chi Minh city, Vietnam; 2 Pham Ngoc Thach Hospital, Ho Chi Minh City, Ho Chi Minh, Vietnam; 3 Nuffield Department of Medicine, Centre for Tropical Medicine and Global Health, University of Oxford, Oxford, England, UK

**Keywords:** tuberculosis, sputum digestion, sputum decontamination, sputum frozen storage

## Abstract

**Background:** Collecting and storing large number of sputum samples with a view to culturing these in the future requires an efficient initial handling method. We devised a modified sputum digestion and decontamination method that maximised storage capacity and *Mycobacterium tuberculosis* (M.tb) recovery from culture while minimising laboratory workload and risk of contamination.

**Methods:** We collected smear microscopy positive sputum samples from patients with pulmonary tuberculosis (TB). The sputum samples were split and processed using both the standard N-Acetyl-L-cysteine and sodium hydroxide (NALC-NaOH) method and our modified method before freezing and later culturing in BD BACTEC 960 Mycobacterium Growth Indicator Tubes (MGIT) system. We assessed the Time to Positivity (TPP) and Growth Unit (GU) data.

**Results:** We selected 22 sputum samples to compare two digestion and decontamination methods. The samples that underwent the modified method had longer TTP (p < 0.05) but similar GU in comparison to standard method. Overall, 1/22 samples failed to grow in MGIT after being processed by the modified method. We then applied the modified method to 348 sputum samples with Rifampicin resistance detected by GeneXpert MTB/RIF assay, which were frozen for between 1-25 months. The overall MGIT positive, negative, and contamination rate was 90.5%, 7.8%, and 1.7%, respectively. There was no significant difference in MGIT result when samples were grouped by duration of storage or positive smear grade.

**Conclusions:** Our modified method yielded acceptable M.tb recovery rate and low contamination risk while allowing us to collect and store thousands of sputum samples over a long period of time for future tests.

## Introduction

The diagnosis of tuberculosis (TB) is not always straightforward and some clinical samples are easier to obtain than others ( Harries & Kumar, 2018). Studies in patients with pulmonary TB who can produce sputum samples benefit from cheap and non-invasive sampling. However, for studies that require large numbers of sputum samples to be collected, significant sample processing and storage capacity may be needed. Furthermore, where it is only determined after sample storage which samples require further testing, an efficient initial handling process is required. Samples need to be transferred to volume efficient containers (freezer vials), maximising the chances of obtaining a *Mycobacterium tuberculosis* ( *M.tb*) culture at a later date, whilst avoiding cross-contamination and minimising the risk to laboratory staff. Storage without prior decontamination has been attempted previously with some success ( Shinu *et al.*, 2016; Tessema *et al.*, 2011). However, these studies did not concentrate primary sputum samples into volume efficient containers or use cryopreservative such as glycerol for long term storage of bacteria. There is thus room for logistical improvement and potentially for higher positive culture yields through the use of cryopreservatives ( Shu *et al.*, 2012).

We describe a modified method for decontaminating and storing sputum samples that can be applied for studies which process large numbers of clinical samples daily and selectively culture over an extended period of time.

## Methods

### Ethical approval

The study was approved by the Institutional Research Board of Pham Ngoc Thach Hospital as the supervisory institution of the District Tuberculosis Units (DTUs) in Ho Chi Minh City, Vietnam (CS/PNT/20/01) and the University of Oxford (OxTREC ref 51-19), UK. IRB approval was granted on February 10th, 2020, and data collection began on March 2nd, 2020.

For initial collection of sputum samples, we specifically requested a waiver from the IRB for consent from patients we screen, and from whom we collect and store samples.

We obtained written informed consent from patients who went on to be recruited into the study. As all patients with multidrug resistance TB (MDR-TB) were recruited into the study, we obtained written consent from these patients. The other samples contributing to this manuscript were selected randomly from the archived anonymous sputum samples, which the IRB waived the need of consent for.

### Study population and biological samples

This was a prospective observational cohort study in Ho Chi Minh city, Vietnam between 1 ^st^ March 2020 and 31 ^st^ August 2023 (42 months). All related data were collected on recruited patients within the aforementioned period, with patient outcomes and medication changes recorded for a further 12 months thereafter for patients still on treatment at 42 months. The screening population was adult patients (18 years or older) with a smear microscopy positive clinical sample or had *M. tuberculosis* detected by MTB/RIF Xpert with the expected number of about 30,000 samples. Sputum samples were collected in 50-mL Falcon tubes from all screenings from 1 ^st^ March 2020. A pilot study was conducted using sputum samples collected in the first week after commencement. We adopted the modified sputum processing procedure after the results were finalized. The screening patients were recruited to MDR-TB transmission study with consent form if they had both *M. tuberculosis* detected and Rifampicin resistance detected by MTB/RIF Xpert.

### Direct culture vs. culture from frozen samples

A total of 22 samples were selected randomly for a pilot experiment. No changes were made following the pilot. N-Acetyl-L-cysteine (NALC)-NaOH 2% was added to each sputum sample in a 10:1 ratio before vortexing. The liquefied samples were divided into two equal aliquots. One aliquot was processed with standard method, whereby the sample was incubated further at room temperature (RT) for up to 20 minutes, then neutralized by adding up to 40 mL phosphate buffered saline (PBS), and centrifuged at 3,220xg for a further 20 minutes. The supernatant was removed and the pellet cultured in BD BACTEC Mycobacterial Growth Indicator Tube 960 (MGIT 960, Becton Dickinson), with PANTA supplement, a cocktail of antibiotic (polymyxin-B, amphotericin-B, nalidixic acid, trimethoprim, azlocillin). The second aliquot was processed with a modified method that neutralized the sample with PBS immediately and centrifuged at 3,220xg for 20 minutes. The supernatant was removed and PBS containing 20% glycerol was added to the remaining pellet in a 1:1 volume ratio before freezing the samples at -80°C in 0.5 mL screw-capped tubes. The frozen samples were thawed after 7 days before being transferred to a fresh sterile 1.5 mL Eppendorf tube and mixed with 1 mL NALC-NaOH 2% solution. After incubating for 20 minutes at RT, samples were centrifuged using microcentrifuges at 11,000xg for 90 seconds. The supernatant was then removed and cultured in MGIT with PANTA supplement.

### Culture from samples frozen over longer time periods

Based on the pilot, we processed and froze all prospectively collected samples as described. After two years of collecting sputum samples, we needed to culture all samples for which rifampicin resistance had been detected by the GeneXpert MTB/RIF assay for the MDR-TB transmission study. These samples were all defrosted and processed according to the same method. Although we had no opportunity to compare growth to corresponding aliquots that had not been frozen in long-term storage, these samples allowed us to assess the impact of duration of freezing on mycobacterial growth.

### Data collection

For each culture, MGIT culture vial was used in combination with BD BACTEC 960 system, which generated three variables of interest: “Time to positive” (TTP) in unit of days and hours (DD;HH), “Growth Unit” (GU), and MGIT result of “Positive” and “Negative”. TTP unit was converted into hours while GU was analysed as generated. MGIT results were classified into “Positive” and “Negative” as generated by the system. The threshold for MGIT result of “Negative” was 42 days without any changes in GU as detected by the system; any increase in GU in less than 42 days were detected as “Positive”. “Contaminated” MGIT result was added when a culture with “Positive” MGIT result showed TTP of less than three days and/or the medium showed turbidity without presence of small grains or granules. The duration of storage at -80°C for each sample was the difference in months between sample processing date and start of culture date and the Ziehl-Neelsen smear grade was recorded.

### Statistical analysis

All statistical analysis was performed with RStudio (RRID:SCR_000432) version 1.2.5019 using R Project for Statistical Computing (RRID:SCR_001905) version 4.2.2. The Wilcoxon signed-rank test was performed to compare data from two processing methods and Pearson’s Chi-squared test was used to analyse the relationship between MGIT culture result *versus* smear microscopy grade or duration of frozen storage.

## Results

### Direct culture vs. culture from frozen samples

**Figure 1.**
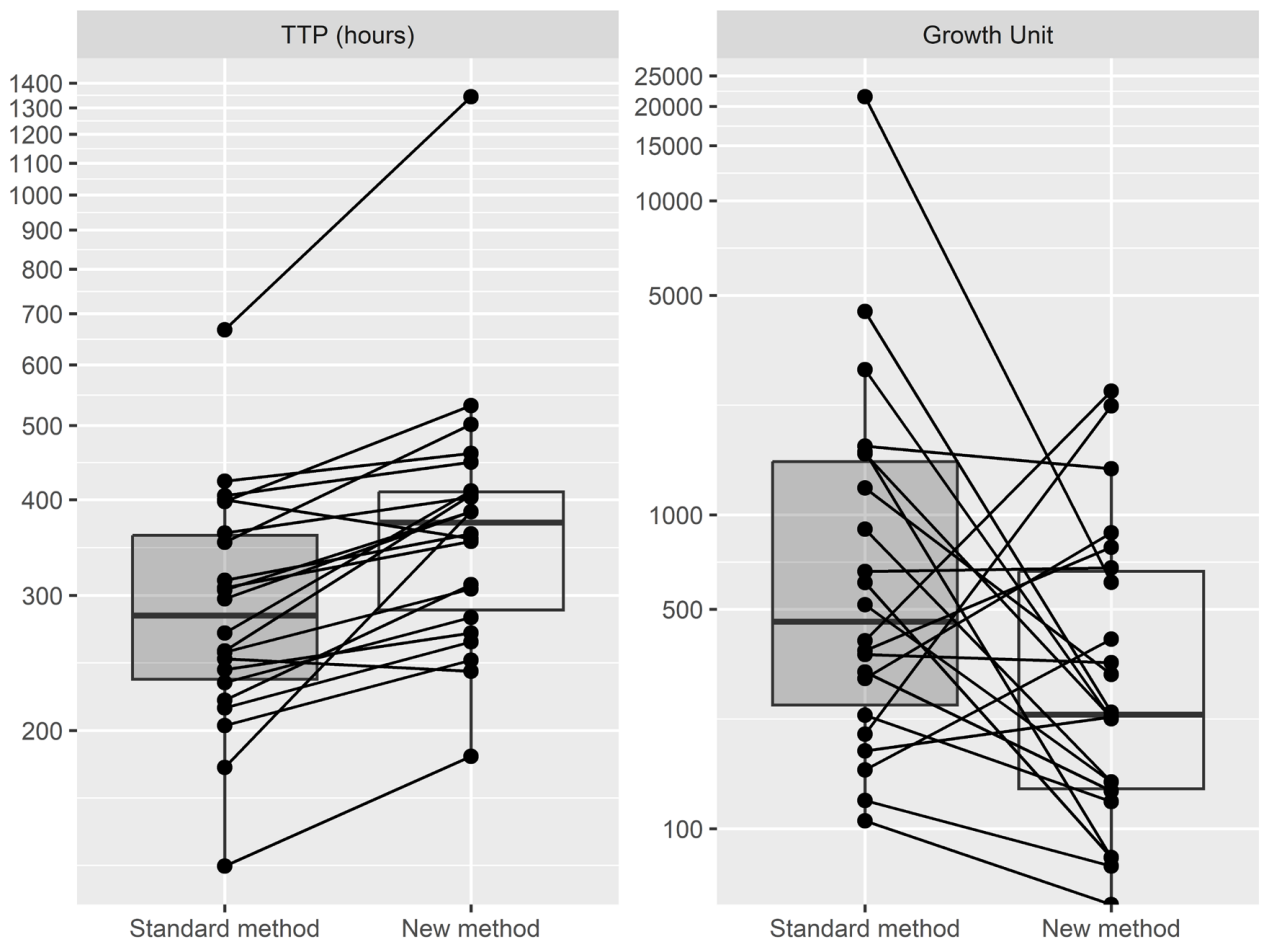
TTP in hours (Left) and Growth Unit (Right) reported by MGIT system between Standard method and New method (n=22). Standard method decontaminate sputum samples using NALC-NaOH 2% and cultured immediately); New method concentrates sputum samples with brief exposure to NALC-NaOH 2%, frozen in 7 days before decontamination using NALC-NaOH 2% and cultured. The vertical lines represent the tails of the boxplot. TTP, Time to positive; MGIT, Mycobacterium Growth Indicator Tubes; NALC-NaOH, N-Acetyl-L-cysteine and sodium hydroxide.

To test how our modified method of sputum decontamination and frozen storage affects the MGIT culture, 22 sputum samples were divided into two aliquots and processed by two methods ( Figure 1) ( Nguyen Le, 2023). We compared the growth units from the MGIT system, an indirect measurement of microbial aerobic growth, and time-to-positivity, the amount of time for a MGIT culture to reach predefined growth units. All 22 aliquots that were decontaminated and cultured immediately grew in MGIT culture, along with 21/22 to which the modified method was applied. Those aliquots that were decontaminated and cultured immediately showed a mean TTP of 303 ± 113 hours and estimated mean growth units of 1,830 ± 4,515 unit. The mean TTP and GU after the modified method was applied was 401 ± 228 hours and 536 ± 677 units, respectively. The Wilcoxon signed-rank test showed that the TTP was significantly different between the two methods in which the immediate decontamination and culture of the sputum showed faster TTP (p=0.0001), while there was no significant difference in terms of growth units (p > 0.05).

### Culture from samples frozen over longer time periods

A total of 348 rifampicin resistant frozen sputum concentrates collected over a two year period were selected for culture. Overall, 90.5% were positive in MGIT culture, 7.8% were negative, and 1.7% were contaminated. Table 1 shows samples grouped by the duration they were frozen at -80°C in months. No significant difference was seen among groups (p > 0.05).

**Table 1.** Number of MGIT samples grouped by duration of storage at -80°C (months) and the corresponding cultures result (%). MGIT, Mycobacterium Growth Indicator Tubes.

# of months stored at -80°C	Culture positive	Culture negative	Contaminated	Total
**1**	34 (82.9%)	7 (17.1%)	0	41
**2**	38 (84.4%)	7 (15.6%)	0	45
**3**	20 (95.2%)	1 (4.8%)	0	21
**4**	2 (100%)	0	0	2
**5**	1 (100%)	0	0	1
**7**	15 (88.2%)	2 (11.8%)	0	17
**8**	25 (100%)	0	0	25
**9**	10 (100%)	0	0	10
**10**	9 (90%)	0	1 (10%)	10
**11**	17 (94.4%)	1 (5.6%)	0	18
**12**	12 (80%)	2 (13.3%)	1 (6.7%)	15
**13**	14 (100%)	0	0	14
**14**	24 (92.3%)	1 (3.9%)	1 (3.9%)	26
**15**	4 (100%)	0	0	4
**16**	16 (100%)	0	0	16
**17**	16 (84.2%)	2 (10.5%)	1 (5.3%)	19
**18**	21 (95.5%)	1 (4.6%)	0	22
**19**	9 (100%)	0	0	9
**20**	10 (90.9%)	0	1 (9.1%)	11
**21**	14 (82.4%)	3 (17.7%)	0	17
**22**	2 (66.7%)	0	1 (33.3%)	3
**24**	1 (100%)	0	0	1
**25**	1 (100%)	0	0	1
**Total**	315 (90.5%)	27 (7.8%)	6 (1.7%)	348

The samples were further categorized by their smear microscopy grade ( Table 2). The proportion positive, negative, and contaminated ranged between 86.5–96.3%, 3.7–11.7%, and 1.8–2.6%, respectively. There was no relationship between smear microscopy grade and MGIT culture result (p > 0.05).

**Table 2.** MGIT positive, negative, and contamination percentage classified by ZN grade (N = 348). MGIT, Mycobacterium Growth Indicator Tubes.

ZN grade	Positive	Negative	Contamination	Total
**3+**	79 (96.3 %)	3 (3.7 %)	0	82
**2+**	96 (86.5 %)	13 (11.7 %)	2 (1.8 %)	111
**1+**	140 (90.3 %)	11 (7.1 %)	4 (2.6 %)	155
**Total**	**315 (90.5 %)**	**27 (7.8 %)**	**6 (1.7 %)**	**348**

## Discussion

We designed a method for processing and freezing large numbers of sputum samples from patients diagnosed with TB. Large observational and randomised studies may require the storage of primary samples with the option of retrieving and culturing them at a later date. Culturing each sample upfront is prohibitively expensive whilst freezing primary samples without any decontamination step risks contamination of future cultures. Our approach uses NALC-NaOH digestion-decontamination to concentrate the mycobacteria within each sample ( Ratnam & March, 1986), before and after freezing, allowing a greater number of sputum samples to be stored in a freezer in 0.5-mL cryovials, and minimising the number of contaminated cultures after defrosting.

We saw that the freezing process extends the time to culture positivity compared to culture without a prior freezing step, and also reduced the growth units reported by the BACTEC MGIT system. Such detrimental effect from freezing sputum was also observed for other microbes such as *Pseudomonas aeruginosa* ( Poonja *et al.*, 2017). Although only one of our 22 samples failed to grow entirely after defrosting, freeze-thawing can lead to some samples not growing in culture ( Shinu *et al.*, 2016; Shu *et al.*, 2012). Our method is not suitable for studies that assay time-to-positivity, but it may be helpful to those needing to store samples first and perform phenotypic drug susceptibility testing or molecular tests on a to-be-defined subset of samples at a later date. Our method works well when applied to 1+, 2+, and 3+ smear microscopy positive samples.

As seen elsewhere, we found that the duration of storage at -80°C did not affect MGIT positivity rate ( Tessema *et al.*, 2011). Our method found an overall MGIT positivity rate of 90.5%, comparing favourably with a 2016 study that found an 86.0% MGIT positivity rate for sputum frozen at -80°C for 3 months ( Shinu *et al.*, 2016). Although the vast majority of the sputum samples were taken before the patients started their treatment, it is possible that a very small number had already started anti-tuberculosis treatment by the time we obtained a sample. We do not have data on how many, but it is clearly the case that prior treatment risks negative sputum cultures. The overall contamination rate resulted from the data was also lower than that of the study in 2016 (1.7% *vs.* 5.2%) ( Shinu *et al.*, 2016) suggesting that modified method of decontamination and storing sputum may reduce contamination risk.

Limitations of our study were that we did not compare alternative methods of decontamination and storage to our own. We instead devised a method that would suit our purposes and local capacity and compared to direct-from-sample culture. This setup gave us confidence to continue with our method, which we then assessed again for the effect of time spent in the freezer on the success of future attempts at culture. We cannot therefore say that our method is better than other methods. It did however yield acceptable outcomes in terms of both culture and contamination. It is also easy and cheap to implement and has allowed us to so far collect and store over 10,000 sputum samples, some for over two years now.

## Data Availability

Zenodo: A modified decontamination and storage method for sputum from patients with tuberculosis. https://doi.org/10.5281/zenodo.7584298 ( Nguyen Le, 2023). This project contains the following underlying data: “22_samples_TTP_GU_method_comparison_dataset.csv" (dataset for comparing standard method and modified method using 22 sputum samples before being cultured in MGIT. MGIT is used in BD BACTEC 960 MGIT system which generates "Time to positive" hours for a culture to growth and "Growth Unit" for estimating the amount of growth.) "348_samples_TTP_GU_modified method_dataset.csv" (dataset for applying modified method on selected 348 sputum samples for culture in MGIT. The dataset contains "Time to positive", "Growth Unit", "ZN smear grade", and "Duration of frozen”.) Data are available under the terms of the Creative Commons Attribution 4.0 International license (CC-BY 4.0).
