# Supplementary material for: A modified decontamination and storage method for sputum from patients with tuberculosis
Source: Wellcome Open Res. 2025 Feb 28;8:166. Originally published 2023 Apr 13. [Version 3] doi: 10.12688/wellcomeopenres.18888.3 (PMC11358684; doi:10.12688/wellcomeopenres.18888.3)
Supplement: Peer review history

# Reviewer report: Reviewer response for version 2

**DOI:** 10.21956/wellcomeopenres.23187.r94424

**Published:** 2024-08-28

**Authors:** Virasakdi Chongsuvivatwong

**Affiliations:** 1 Prince of Songkla University, Kanjanavanich Rd, Kho Hong Hat Yai, Thailand

The first part of the study having 22 specimens divided into 2 aliquots suffers from smallness of the sample size.

 The second part of the study have larger sample size suffers from lacking of standard procedure control. This part showed that the results did not perfectly agree with ZN and Xpert methods. Whether the discrepancy was due to non-TB sputum or deterioration of viability of M.tb is not known.

Is the rationale for developing the new method (or application) clearly explained?

Yes

Is the description of the method technically sound?

Partly

Are the conclusions about the method and its performance adequately supported by the findings presented in the article?

Partly

If any results are presented, are all the source data underlying the results available to ensure full reproducibility?

Yes

Are sufficient details provided to allow replication of the method development and its use by others?

Yes

Reviewer Expertise:

TB research training.

I confirm that I have read this submission and believe that I have an appropriate level of expertise to confirm that it is of an acceptable scientific standard, however I have significant reservations, as outlined above.

# Response

**Published:** 2024-10-01

**Authors:** Quang Nguyen Le

**Affiliations:** OUCRU, Vietnam

Responses in red Reviewer 3:

 The first part of the study having 22 specimens divided into 2 aliquots suffers from smallness of the sample size.

 The second part of the study have larger sample size suffers from lacking of standard procedure control. This part showed that the results did not perfectly agree with ZN and Xpert methods. Whether the discrepancy was due to non-TB sputum or deterioration of viability of M.tb is not known.

 Response: Thank you for your comments. We agree with all you say. We have added a line to the discussion to indicate as a limitation that the pilot study was small. The absence of a control group for the second study was already addressed as a limitation in response to the previous reviewer. Please see the final paragraph of the discussion where we highlight this as an issue. However, for our purposes this was not what was centrally important. What was key was that our simple, pragmatic method yielded an acceptable proportion of positive cultures, which it did. Moreover, we were able to show that the time in the freezer had little impact on culture-ability. We cannot now go back and re-do any of the experiments, so we are left having just to highlight the limitations in the discussion. We hope this is acceptable. Many thanks. Regarding the culture negative samples, the reviewer is again correct. We cannot know whether there were viable Mtb bacilli in the sample to start with, or not.

**Is the rationale for developing the new method (or application) clearly explained?** Yes

**Is the description of the method technically sound?** Partly

 We would appreciate guidance on which bit of the description is not technically sound and we can update, as needed. Thank you.

**Are sufficient details provided to allow replication of the method development and its use by others?** Yes

**If any results are presented, are all the source data underlying the results available to ensure full reproducibility?** Yes

**Are the conclusions about the method and its performance adequately supported by the findings presented in the article?** Partly

 Again, please let us know which conclusions are not adequately supported, and we can update. We felt we were very cautious in our conclusions. Please see the final paragraph. Many thanks.

# Reviewer report: Reviewer response for version 2

**DOI:** 10.21956/wellcomeopenres.23187.r74935

**Published:** 2024-08-27

**Authors:** Irina Kontsevaya

**Affiliations:** 1 Imperial College London, London, England, UK

I have no further comments.

Is the rationale for developing the new method (or application) clearly explained?

Yes

Is the description of the method technically sound?

Yes

Are the conclusions about the method and its performance adequately supported by the findings presented in the article?

Partly

If any results are presented, are all the source data underlying the results available to ensure full reproducibility?

Yes

Are sufficient details provided to allow replication of the method development and its use by others?

Yes

Reviewer Expertise:

Laboratory diagnostics of tuberculosis, biomarkers of tuberculosis disease and cure, treatment monitoring tools.

I confirm that I have read this submission and believe that I have an appropriate level of expertise to confirm that it is of an acceptable scientific standard.

# Reviewer report: Reviewer response for version 1

**DOI:** 10.21956/wellcomeopenres.20944.r69570

**Published:** 2023-11-21

**Authors:** Irina Kontsevaya

**Affiliations:** 1 Imperial College London, London, England, UK

The manuscript presents the results of a two-phase study of evaluation of a modified method for decontamination and storage of sputum samples collected from individuals with pulmonary tuberculosis. While in routine diagnostic it is essential to process and culture sputum samples as soon as possible for fast and precise diagnostics of tuberculosis, storage of sputum samples is often needed in longitudinal studies and randomised clinical trials where culture results might only be needed for a selected sample which cannot be determined at the time of sample collection. A method for storage of sputum in small volumes (cryovials) that does not affect the viability of Mycobacterium tuberculosis in sputum is therefore urgently needed.

 The method proposed in this study is simple, it does not require additional reagents beside those used in the standard procedure, and not time-consuming. It offers a possibility to store samples in small 0.5 ml vials which is important in terms of storage space in the freezers. The study demonstrates that viability and culturability of the sputum samples after applying the modified method is not affected by freezing-thawing and also doesn’t increase the contamination rate. The duration of storage also seems not to affect the MGIT positivity rate. However, it is demonstrated that storage extends the time to culture positivity and reduces growth units. Samples processed with this modified method therefore cannot be used in studies for quantitative evaluation of growth but rather in those where, for example, culture is further used for genomic studies or assessment of bacterial resistance profile.

 Overall, this manuscript is of great interest for researchers conducting large studies in tuberculosis, including randomised controlled trials. However, the limitations of this study do not allow to use it as a recommendation to immediately implement the method in practice. While this can be considered a proof-of-concept, I believe further studies are needed to address the current limitations and multiple further questions that arise from this study. Below is the number of suggested further studies:

1. Direct culture vs. culture from samples frozen for a long period of time. Currently, only 22 samples were compared being cultured directly and after being frozen for 1 week. No such comparison was made for samples frozen for longer periods of time.
2. Effects of storage for longer periods, up to 5-10 years. Long-term storage is important for some types of studies, for example transmission studies on historical samples.
3. Evaluation of the method with storing samples at -20. In the study all samples were frozen at -80. In reality, many studies in tuberculosis are conducted in resource-limited settings where sputum is collected and stored at local clinics and laboratories. In such settings, -80 freezers are often not available. Therefore, in practical terms it would be great to have a method that would allow effective storage of samples at -20 or even at +4.
4. Comparison of the proposed method with other methods of sputum storage. While it is demonstrated that this method is effective and not resource-intensive, other methods for sputum storage can also be characterised this way. To choose the best method to apply in a particular setting, comparison data are needed.

While I appreciate that these suggested studies cannot be implemented in the current manuscript, perhaps the authors could think about further studies to validate their method or motivate other groups to join the evaluation of the method to explore its feasibility.

Is the rationale for developing the new method (or application) clearly explained?

Yes

Is the description of the method technically sound?

Yes

Are the conclusions about the method and its performance adequately supported by the findings presented in the article?

Partly

If any results are presented, are all the source data underlying the results available to ensure full reproducibility?

Yes

Are sufficient details provided to allow replication of the method development and its use by others?

Yes

Reviewer Expertise:

Laboratory diagnostics of tuberculosis, biomarkers of tuberculosis disease and cure, treatment monitoring tools.

I confirm that I have read this submission and believe that I have an appropriate level of expertise to confirm that it is of an acceptable scientific standard.

# Reviewer report: Reviewer response for version 1

**DOI:** 10.21956/wellcomeopenres.20944.r69006

**Published:** 2023-11-16

**Authors:** Bhushan J. Toley

**Affiliations:** 1 Indian Institute of Science, Bengaluru, Karnataka, India

In tuberculosis (TB) diagnostics and research, the storage and subsequent culturing of sputum samples are fundamental yet challenging tasks, especially under resource-constrained conditions or when dealing with high sample volumes. The study presented here introduces a modified method for the handling and preservation of sputum samples containing Mycobacterium tuberculosis with the aim of enhancing their viability over extended storage periods.

 The standard practice of using glycerol as a cryoprotectant is maintained in this method, which is not novel in itself. However, the study proposes a second decontamination step post-thaw as a slight modification to the conventional process. This additional step is posited to potentially improve the recovery of viable bacteria after the samples have been frozen and then thawed for later use.

 While the degree of innovation in the proposed method may not be substantial, it represents an incremental advance that could have practical implications for the storage duration of sputum samples. Such a modification could indeed prove beneficial in settings where the immediate processing of samples is not possible and long-term storage becomes a necessity.

 This paper thus offers a potential improvement in TB sample management, albeit a modest one, which warrants attention for its possible impact on TB management protocols, especially in settings facing logistic challenges in sample processing.

 I have reviewed the dataset and methodology and wish to present a series of observations that merit consideration. These insights aim to refine the research approach and enhance the validity of the findings. They are presented with the intent of constructively supporting the study's progression towards robust, reproducible results.

**Control Groups and Comparative Analysis:**

 The study presents a novel two-step NALC-NaOH decontamination process for sputum samples. Yet, the absence of a direct comparison with a single-step decontamination control group undermines the validation of this method. To substantiate the necessity and effectiveness of the second decontamination step, it is recommended that the authors include such a control group in their analysis.

 Additionally, exploring the viability of samples stored at varied temperatures—room temperature, 4°C, -20°C, as well as -80°C—could provide valuable insights into more resource-efficient storage methods. This would be particularly relevant in settings where maintaining -80°C storage is challenging.

**Statistical and Long-term Storage Experiment’s Shortcomings:**

 The statistical analysis in this study is insufficient, particularly for samples stored over 13 months, potentially skewing conclusions. Specifically, there are a larger number of samples used at lower time points and much smaller number of samples used for later time points. It is critical to adopt a more rigorous statistical model that accounts for variable sample sizes across different storage times. The claim that 90.5% of samples were viable after 25 months should be interpreted with caution. A balanced evaluation of sample viability across varied storage periods is necessary to convincingly determine the method's efficacy for long-term storage.

**Reproducibility and Robustness:**

 Although not a pressing issue, the reproducibility of the modified method when applied in different laboratory environments or by different technicians may be addressed in future studies. Ensuring reproducibility is key to the method's robustness and broader application. Future research could enhance the study's validity by providing such reproducibility data.

**Data Presentation:**

 The data presentation could be optimized for clarity: integrating median lines into the boxplots would provide a clear depiction of central tendencies, while simplifying TTP and GU connections with lighter lines would reduce clutter. Additionally, distinctly illustrating whiskers on boxplots would elucidate data variability and outliers, improving overall interpretability.

**Literature Context:**

 The discussion would gain depth from a comparison of the modified method against other methods cited in current literature. This broader perspective would help situate the study within the existing research landscape.

**Conclusion and final comments**:

 While the study introduces a promising technique potentially beneficial for tuberculosis diagnostic practices, the manuscript currently falls short in demonstrating the full validity of the proposed method due to the lack of proper control groups and robust statistical analysis. I recommend significant revisions to address these gaps before the manuscript can be considered for acceptance. The enhancements proposed here would not only strengthen the scientific rigor of the paper but also its contribution to the field.

Is the rationale for developing the new method (or application) clearly explained?

Yes

Is the description of the method technically sound?

Yes

Are the conclusions about the method and its performance adequately supported by the findings presented in the article?

Partly

If any results are presented, are all the source data underlying the results available to ensure full reproducibility?

Partly

Are sufficient details provided to allow replication of the method development and its use by others?

Yes

Reviewer Expertise:

Point of care medical daignostics.

I confirm that I have read this submission and believe that I have an appropriate level of expertise to confirm that it is of an acceptable scientific standard, however I have significant reservations, as outlined above.

# Response

**Published:** 2024-01-25

**Authors:** Quang Nguyen Le

**Affiliations:** OUCRU, Vietnam

*Control Groups and Comparative Analysis: The study presents a novel two-step NALC-NaOH decontamination process for sputum samples. Yet, the absence of a direct comparison with a single-step decontamination control group undermines the validation of this method. To substantiate the necessity and effectiveness of the second decontamination step, it is recommended that the authors include such a control group in their analysis. Additionally, exploring the viability of samples stored at varied temperatures—room temperature, 4°C, -20°C, as well as -80°C—could provide valuable insights into more resource-efficient storage methods. This would be particularly relevant in settings where maintaining -80°C storage is challenging.*

 We thank the reviewer for the thoughtful review and detailed comments. Our study consisted of two phases: a pilot to compare one-stage and two-stage procedures, and a later stage to look at the impact on the ability to culture over longer periods of time. As part of the pilot study we therefore did have a control group. As the comparison between standard and new methods was very similar in the pilot phase, the next step was to look at samples that had been frozen for longer periods of time. We were unable to include a further control group here as the samples had already been frozen (this was retrospective). As we conducted this small study to inform how we would handle a large number of samples for an observational study that was about to start, we did not have time to collect several hundred samples prospectively and aliquot these into experimental and control groups. As the main study is coming to an end, we do not have the resource now to repeat this experiment. However, we have included a section in the discussion to acknowledge this limitation. The same goes for the idea that we repeat the experiment at different temperatures. It’s a good idea, but unfortunately not possible as part of this little project.    

*Statistical and Long-term Storage Experiment’s Shortcomings: The statistical analysis in this study is insufficient, particularly for samples stored over 13 months, potentially skewing conclusions. Specifically, there are a larger number of samples used at lower time points and much smaller number of samples used for later time points. It is critical to adopt a more rigorous statistical model that accounts for variable sample sizes across different storage times. The claim that 90.5% of samples were viable after 25 months should be interpreted with caution. A balanced evaluation of sample viability across varied storage periods is necessary to convincingly determine the method's efficacy for long-term storage. * 

 The current text does not actually state that 90.5% were viable after 25 months. It says “Overall, 90.5% were positive in MGIT…”, which is true. In the discussion we later state that the overall positivity rate was 90.5% and compare this with the positivity rate at 3 months seen by Shinu et. al.. If anything, our results would have been even better had we cultured them at 3 months and not included such a long tail. So the skew is unlikely introducing bias when it comes to that conclusion. However, we agree that “No significant difference was seen among groups (p > 0.05)” is an inadequate analysis. We have therefore added a figure that shows the mean percentage of samples (plus 95% confidence interval) that were positive at each month, together with the number of samples frozen for each period of time. The figure summarises the output of a logistic regression model that uses natural cubic splines to account for the non-linear trend over time. MGIT status (pos/neg) at each month is the dependent variable, and the number of months frozen for each month is the independent co-variable. This shows that a greater percentage of our samples were positive when compared to Shinu et al.    

*Reproducibility and Robustness: Although not a pressing issue, the reproducibility of the modified method when applied in different laboratory environments or by different technicians may be addressed in future studies. Ensuring reproducibility is key to the method's robustness and broader application. Future research could enhance the study's validity by providing such reproducibility data.*

 We agree and have added a sentence to the discussion to highlight this.     

*Data Presentation: The data presentation could be optimized for clarity: integrating median lines into the boxplots would provide a clear depiction of central tendencies, while simplifying TTP and GU connections with lighter lines would reduce clutter. Additionally, distinctly illustrating whiskers on boxplots would elucidate data variability and outliers, improving overall interpretability.*

 Done.  

*Literature Context: The discussion would gain depth from a comparison of the modified method against other methods cited in current literature. This broader perspective would help situate the study within the existing research landscape.*

 We have edited the discussion a little but there aren’t additional papers that are directly comparable to our that we were able to find.  

*Conclusion and final comments: While the study introduces a promising technique potentially beneficial for tuberculosis diagnostic practices, the manuscript currently falls short in demonstrating the full validity of the proposed method due to the lack of proper control groups and robust statistical analysis. I recommend significant revisions to address these gaps before the manuscript can be considered for acceptance. The enhancements proposed here would not only strengthen the scientific rigor of the paper but also its contribution to the field.*

 Thank you. We have done our best to address your points. We hope our answers are clear and satisfactory.
